# The 4 F (Fat, Fascia, Fibrin, and Fat) Technique for Skull Base Reconstruction in Endoscopic Transorbital Surgery

**DOI:** 10.1007/s00701-025-06667-5

**Published:** 2025-09-29

**Authors:** Sergio Corvino, Francesco Corrivetti, Giuseppe Catapano, Giuseppe Corazzelli, Antonio Colamaria, Edisher Maghalashvili, Iacopo Dallan, Domenico Di Maria, Germano Di Matteo, Giorgio Iaconetta, Matteo de Notaris

**Affiliations:** 1 Department of Neurosciences, Reproductive and Odontostomatological Sciences, School of Medicine, Neurosurgical Clinic, University of Naples “Federico II”, 80131 Naples, Italy; 2 Neuroanatomy Laboratory, European Biomedical Research Institute of Salerno (EBRIS), Salerno, Italy; 3 Department of Neurosurgery, A.O.U. “San Giovanni Di Dio E Ruggi d’Aragona”, Salerno, Italy; 4 Department of Neurosurgery, “Ospedale del Mare”, 80147 Naples, Italy; 5 Department of Neurosurgery, Santa Maria Delle Grazie Hospital, 80078 Naples, Italy; 6 Department of Neurosurgery, “Riuniti”, 71122 Foggia, Italy; 7 Department of Neurosurgery, American Hospital Tbilisi, Tiblisi, Georgia; 8 Unit of Otorhinolaryngology, Azienda Ospedaliero-Universitaria Pisana, Pisa, Italy; 9 Unit of Otorhinolaryngology, BG. Rummo Hospital, Benevento, Italy; 10 Department of Ophthalmology, A.O.U. “San Giovanni Di Dio E Ruggi d’Aragona”, Salerno, Italy

**Keywords:** Endoscopic Transorbital Approach, Skull Base Reconstruction, Complications, CSF leak

## Abstract

**Background:**

Superior eyelid endoscopic transorbital approach (SETOA) has demonstrated broad versatility in addressing heterogeneous lesions involving the paramedian anterior and middle skull base in carefully selected patients. Although various skull base reconstruction techniques have shown promising results in reducing cerebrospinal fluid (CSF) leaks, no standardized method has yet been established that consistently ensures optimal outcomes in the presence of an intraoperative CSF leak to achieve a watertight seal and minimize the risk of potentially life-threatening complications.

**Methods:**

Preliminary data from a monoinstitutional surgical series of patients harboring different intracranial lesions, in whom intraoperative CSF leak was detected and who underwent reconstruction during SETOA using a novel method defined “4F”, were retrospectively analyzed. The technique consists of intradural autologous fat graft, extradural fascia lata, fibrin glue and extradural autologous fat graft. Postoperative functional and esthetic outcome, particularly reconstruction-related complications, were assessed over a follow-up period of 14–38 months.

**Results:**

The surgical series included 16 patients (2 metastases, 1 orbital lymphoma, 10 meningiomas, 2 trigeminal schwannomas, 1 case of postoperative CSF leak). SETOA was performed in 13 cases, while in the remaining three patients an extended lateral rim orbitotomy variant was added. No cases of CSF leak were observed during the follow-up period. The method provided effective reconstruction, with no instances of major or even minor reconstruction-related complications —such as proptosis, enophthalmos, meningoencephalocele, diplopia, new onset ocular paresis or wound infection—and no revision surgeries were required.

**Conclusion:**

This preliminary experience suggests that the 4F reconstruction technique may be a feasible option for managing osteodural defects during SETOA. It accomplishes the goals of skull base reconstruction, to achieve a watertight closure and avoid dead space. However, given the limited sample size and lack of a control group, definitive conclusions cannot be drawn. Further studies with larger cohorts, standardized outcome measures, and comparative methods are required to assess its final clinical utility.

## Introduction

Since the introduction of the Trans-Orbital Neuro-Endoscopy Surgery (TONES) concept in 2010 by Moe et al. [27], the superior eyelid endoscopic transorbital approach (SETOA) has shown wide versatility, effectiveness and safety in addressing heterogeneous intracranial lesions involving the paramedian anterior and middle skull base, in selected patients, thanks to its peculiar advantages [4, 6–10, 12, 13, 15, 19–22, 24, 25, 27, 29–31]. As for any new technique, its full potential has yet to be revealed and several anatomical studies and surgical series continuously report refinements regarding the three main steps of the procedure: creation of the surgical corridor and exposure of the lesion [6–8, 12, 23, 28], removal of the relevant pathology, and reconstruction of the bony and/or dural defect [11]. In this setting, despite it represents one of the major challenges in skull base surgery, the reconstruction phase must be carefully performed to prevent/decrease the risk of potentially life-threatening complications, such as cerebrospinal fluid (CSF) leakage, meningitis, tension pneumocephalus [9, 14, 17]. Therefore, over the last years, several methods of skull base reconstruction have been described during both endoscopic and open approaches, in tandem with the refinements of the different surgical techniques and the innovation on the heterologous implant materials, but always pursuing the same goals: to ensure a watertight closure to restore the integrity of the barrier between the intra- and extradural compartments and eliminate the dead space [2].

**Fig. 1:**
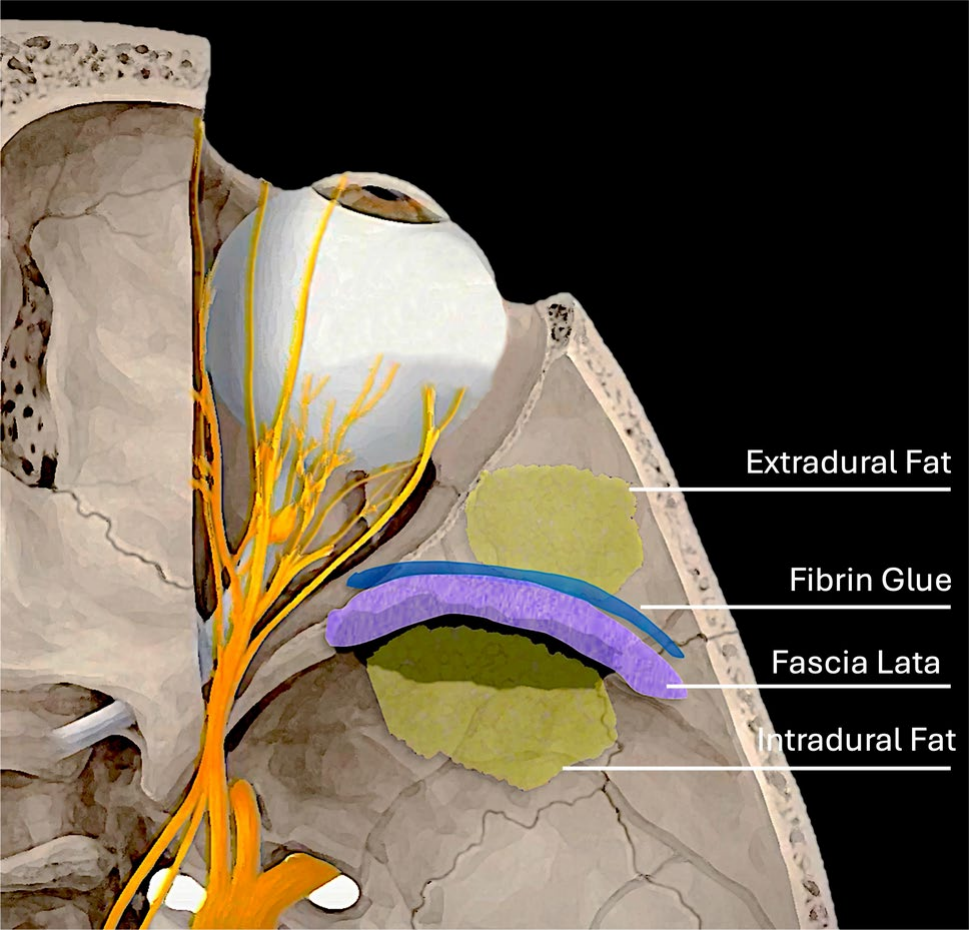
Illustrative draw of 4 F reconstruction technique of right orbit after superior eyelid endoscopic transorbital approach

In the present study we report our preliminary experience using the reconstruction method of the osteodural defect during SETOA in a surgical series that we defined “4F technique”, in which the first “F” refers to the adoption of autologous fat intradurally positioned, the second “F” stands for fascia lata extradurally placed to cover the osteodural defect, the third “F” stands for fibrin glue to fix the previous materials, and the last “F” is used for autologous fat extradurally placed to fill the dead space (Fig. 1). Postoperative functional and esthetic outcomes, with emphasis on reconstruction-related complications, were assessed.

## Methods

### Patient population

Medical record data of patients who underwent skull base reconstruction using the 4 F method during SETOA between January 2022 and January 2024 at the A.O. San Pio (Benevento) and A.O.U. San Giovanni di Dio e Ruggi D’Aragona (Salerno) and operated by the senior author MdN, were retrospectively reviewed. Inclusion criteria were pathologies requiring deliberate dural opening and/or presence of CSF leak, adult population (> 18 years), complete medical record data (clinical, neuroradiological, surgical and outcome data).

Clinical (preoperative neurological and ophthalmological assessments), neuroradiological (lesion location and pattern of growth assessed through contrast-enhanced preoperative magnetic resonance imaging), pathological (type of lesion), surgical and outcome (postoperative CSF leak, enophthalmos, proptosis, diplopia, new onset ocular paresis, wound infection) data were analyzed and discussed.

Follow-up ranged from 14 to 38 months.

### Surgical technique

The standard superior eyelid endoscopic transorbital approach to the middle fossa was performed as previously described in the pertinent literature [13]. In these cases, the creation of the surgical corridor required only the drilling of the lateral orbital wall (LOW) between the superior orbital fissure (SOF) and the inferior orbital fissure (IOF) [1].

In cases where an “extended” [12] variation of the approach was adopted, the lateral orbital rim was removed through two osteotomies across the frontozygomatic suture at the beginning of the procedure and then repositioned at the end through screws and titanium plate. Alternatively, an open-door extended variation (Open-Door Endoscopic Transorbital Approach—ODETA) [8] was performed, in which the lateral orbital rim was only displaced in a hinge-orbitotomy based on osteo-pericranial flap and then repositioned it at the end of the procedure.

For intradural lesions, the dura mater was opened according to the specific pathology.

**Fig. 2:**
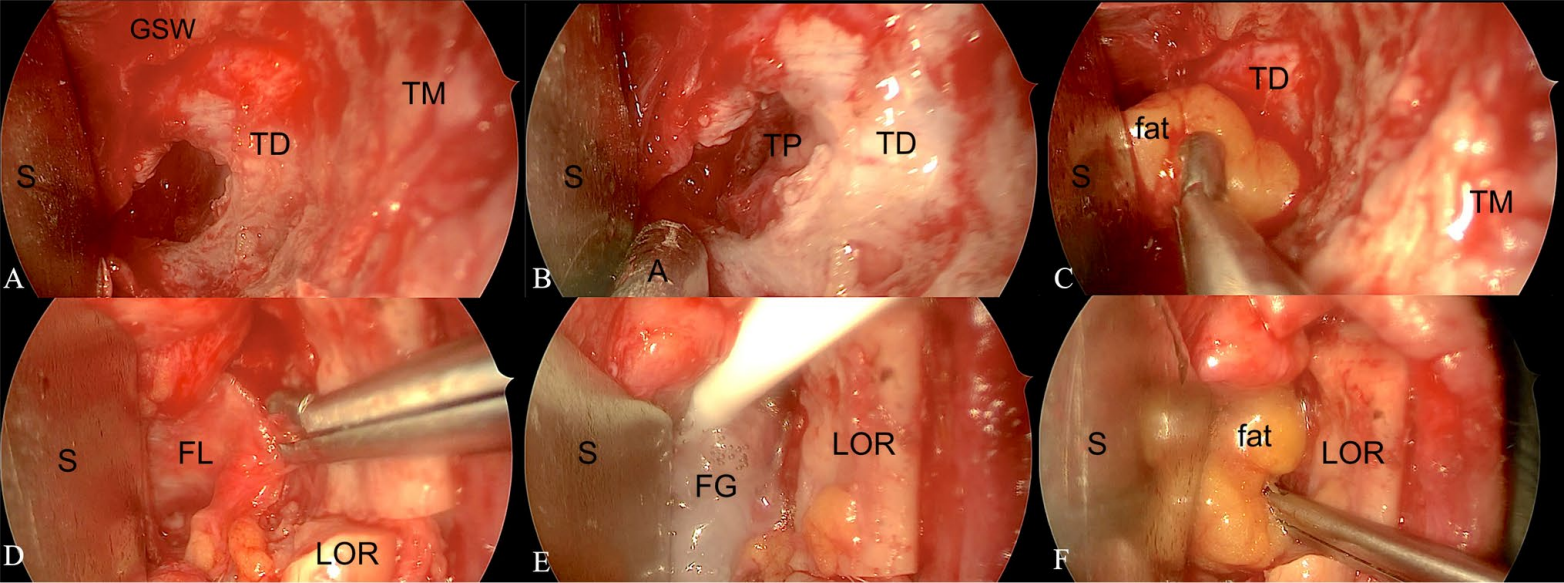
Intraoperative reconstruction of the osteodural defect after SETOA through 4 F technique. Left side. **A-B** Dural defect on the temporal pole dura mater; **C** Intradural fat positioning; **D** Fascia Lata positioning; **E** Thin layer of Fibrin Glue sprayed over the fascia lata; **F** Extradural Fat positioning. (S: Spatula, GSW: Greater Sphenoid Wing; TD; Temporal Dura; TM: Temporalis Muscle; A: Aspirator; TP: Temporal Pole; FL: Fascia Lata; LOR: Lateral Orbital Rim; FG: Fibrin Glue)

In all cases the reconstruction of the osteo-dural defect was performed using the “4F” technique (Fig. 2). In detail, intradural autologous fat graft (first F as “Fat”) was positioned inside the dural (or periorbital in cases of intraorbital lesions) defect; a layer of fascia lata (second F as “Fascia lata”) was positioned extradurally to cover the dural defect; fibrin glue (third “F” stands for “Fibrin glue”) used to fix the previous materials; finally, autologous fat graft (fourth F as “Fat”) was extradurally positioned to fill the dead space between the orbit medially, the temporal pole posteriorly and the temporalis muscle laterally.

As suggested by Moe. [26], at the end of the reconstruction procedure, 2–3 mm of proptosis was intentionally left to account for the expected resolution of the edema over the next 2 weeks which will lead to binocular symmetry. The periosteum was approximated with 5–0 Biosyn sutures, and the orbicularis oculi muscle was sutured with 7–0 Vicryl sutures. The eyelid skin incision was sutured with running 6–0 polypropylene sutures.

## Results

The series consisted of 2 metastases from oropharyngeal cancer (1 located in the parapharyngeal space and 1 in infratemporal fossa), 1 orbital lymphoma, 1 primary and 2 recurrent spheno-orbital meningiomas, 1 petroclival meningioma, 1 temporal pole meningioma, 1 orbital roof meningioma, 3 cavernous sinus meningiomas, 2 trigeminal schwannomas, 1 case of postoperative CSF leak, 1 meningioma of the great sphenoid wing.

The standard SETOA to the middle fossa was performed in thirteen of the sixteen patients. In the remnant three cases, an extended variation of the approach was adopted. In detail, in two patients the lateral orbital rim was removed through two osteotomies and then repositioned, while in one case, an ODETA was performed.

Most lesions were intradural extra-axial (10 meningiomas, 1 CSF leak after giant adenoma exeresis), requiring dural opening for access. In the few cases of extradural lesions (2 metastases, 2 trigeminal schwannomas, 1 orbital lymphoma) intraoperative CSF leak was detected during the tumor removal while attempting to dissect the dura mater from the lesion.

Preoperative clinical evaluation revealed two cases of diplopia and 13 cases of proptosis confirmed on axial CT scan.

**Fig. 3:**
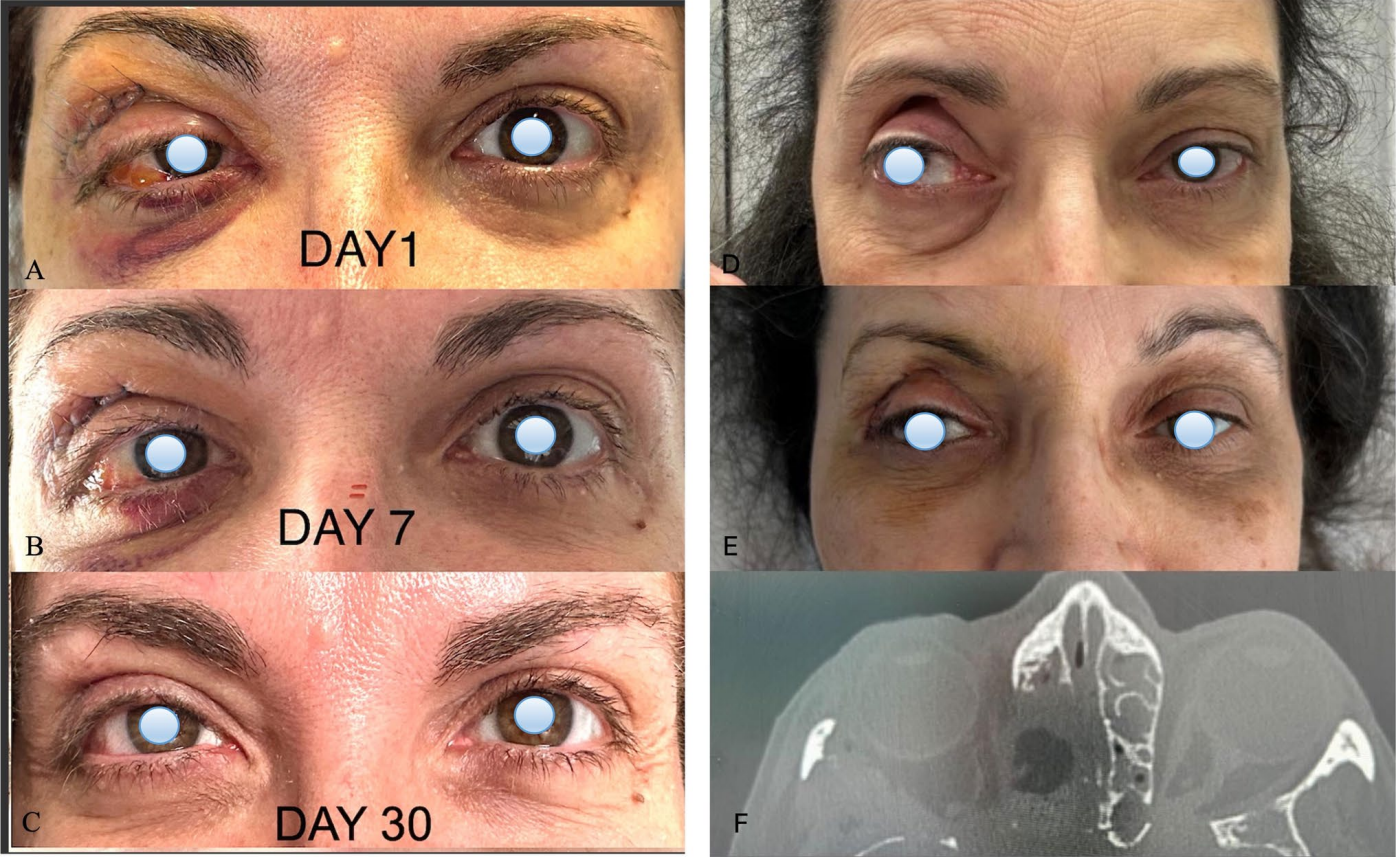
**A-C** Postoperative photograph of a patient underwent superior eyelid endoscopic transorbital approach for right spheno-orbital meningioma causing preoperative proptosis. **A** First day after surgery; **B** 1 week after surgery; **C** 1 month after surgery. **D-F** Postoperative photograph of a patient underwent superior eyelid endoscopic transorbital approach for giant pituitary adenoma (**D-E**) Clinical and (**F**) Radiological resolution of preoperative proptosis

After the surgical procedure and reconstruction with 4 F technique, as expected, mild postoperative iatrogenic proptosis was observed in all patients, but it was clinically resolved within 1 week after discharge and confirmed at first postoperative CT scan evaluation that we routinely performed one month after patient’s discharge (Fig. 3). Both patients with diplopia reported progressive improvement during follow-up. In addition, in regard of the potential reconstruction-related complications, no cases of enophthalmos, nor meningoencephalocele, new onset ocular paresis, wound infection were observed. No cases of postoperative CSF leak were observed, and no revision surgeries were required throughout the entire follow-up period (14–38 months).

All these data are summarized in Table 1.

**Table 1:** Pathological, clinical and outcome data of the surgical series of patient treated by superior eyelid endoscopic transorbital approach and 4 F technique reconstruction

Case	Pathology		Preoperative Status		Postoperative Complications					
	**Type**	**Location**	Proptosis	Diplopia	Proptosis	Enophthalmos	**CSF** **leak**	**Diplopia**	**New onset** **ocular paresis**	**Wound** **Infection**
1	metastasis	Middle TF/Orbit	Yes	No	Transient (resolved 1 week)	No	No	No	No	No
2	metastasis	Middle TF/Orbit	Yes	No	Transient (resolved 1 week)	No	No	No	No	No
3	lymphoma	Orbit	Yes	Yes	Transient (resolved 1 week)	No	No	No	No	No
4	meningioma	Spheno orbital	Yes	Yes	Transient (resolved 1 week)	No	No	No	No	No
5	Recurrent meningioma	Spheno orbital	Yes	No	Transient (resolved 1 week)	No	No	No	No	No
6	Recurrent meningioma	Spheno orbital	Yes	No	Transient (resolved 1 week)	No	No	No	No	No
7	meningioma	Orbital roof	No	No	Transient (resolved 1 week)	No	No	No	No	No
8	meningioma	Petroclival	Yes	No	Transient (resolved 1 week)	No	No	No	No	No
9	meningioma	Temporal pole	Yes	No	Transient (resolved 1 week)	No	No	No	No	No
10	meningioma	Cavernous sinus	Yes	No	Transient (resolved 1 week)	No	No	No	No	No
11	meningioma	Cavernous sinus	Yes	No	Transient (resolved 1 week)	No	No	No	No	No
12	meningioma	Cavernous sinus	Yes	No	Transient (resolved 1 week)	No	No	No	No	No
13	schwannoma	Trigeminal	No	No	Transient (resolved 1 week)	No	No	No	No	No
14	schwannoma	Trigeminal	No	No	Transient (resolved 1 week)	No	No	No	No	No
15	Meningioma	Great Sphenoid Wing	Yes	No	Transient (resolved 1 week)	No	No	No	No	No
16	Postoperative CSF leak (Adenoma)	Middle TF/Orbit	Yes	No	Transient (resolved 1 week)	Yes	No	No	No	No

## Discussion

One of the main advantages of superior eyelid endoscopic transorbital approach, both in its standard and extended variation, is its minimally invasive nature, involving limited bone removal which requires a simple reconstruction at the end of the procedure.

Standard SETOA to middle cranial fossa requires only the drilling of the LOW between SOF and IOF. Concerning the extended endoscopic transorbital approaches, different variants have been described: Noiphithak et al. [28] temporarily remove the lateral orbital rim between the frontozygomatic suture and the zygoma; Lim et al. [23] remove the superior-lateral orbital rim creating a large one-piece bone flap which includes part of the orbital roof; Corvino et al. [8] just displace the lateral orbital rim straddling the frontozygomatic suture but leaving it in situ in a hinge-fashion. In conclusion, even in extended transorbital approaches, the orbital rims are always preserved, therefore the reconstruction step involves only the LOW with the aim to avoid dead space which could otherwise lead to enophthalmos as well as esthetic and functional deficits.

When dealing with intradural pathologies or in cases of dural tears or intraoperative CSF leak, along with the bony defect, the dural defect must also be meticulously repaired with the aim of ensuring a watertight closure to avoid/prevent infectious and potentially life threating diseases.

For this purpose, several methods have been described for repairing the osteodural defect, following the principles provided by the well-consolidate endoscopic endonasal approach which has paved the way, and has achieved an incredible decrease in the rate of CSF leak over the years thanks to the continuous refinements of the technique and materials [16, 17]. In this setting, the skull base endoscopic team of the Neapolitan school, has introduced a pivotal technique, namely “3F” [3], involving autologous *fat* grafting, nasoseptal *flap* coverage and *flash* postoperative patient mobilization, for the skull base reconstruction after extended endoscopic endonasal approaches and achieving excellent results in terms of postoperative complications and clinical outcomes, with a rate of CSF leak of 4%. This method, supported by its results, inspired our technique.

Materials commonly used for the reconstruction after endoscopic transorbital approach include dural substitute, free graft of autologous fat, free graft of autologous fascia lata, fibrin glue, polydiaxanone (PDS) sheet, titanium mesh, adopted in isolated or variously combined manners, as single or multilayers. To date, however, the techniques are different among institutions and well-defined algorithm of reconstruction is missing [5]. Commonly, in presence of a small dural defect and CFS leak, a free fat graft and fibrin glue are sufficient; otherwise, for larger bony dural defect and CSF leak, a multilayer reconstruction, involving free fat graft and dural substitute or fascia lata, are recommended.

A recent review of the literature on the rates of postoperative CSF leak during SETOA for both extra-axial and intra-axial pathologies [5], reports an overall rate of CSF leak of 2.5% (6 cases out of 240 procedures), of which no case of CSF leak after resection of extradural lesions, and very low rates, 3.05% and 6.25%, after resection of intradural extra-axial and intra-axial lesions, respectively. Higher rates of CSF leak were registered after reconstruction with free local flap (50%), followed by dural substitute and fat (4.54%), fascia lata and fat (3.39%) and dural substitutes/fascia lata/muscle free flap (1.2%); no cases of CSF leak were reported after reconstruction with dural substitutes alone, and after dural substitutes/fascia lata/fat. In addition, in a recent retrospective study, Jeon et al. [18] report the data from a monoinstitutional surgical series including heterogeneous lesions affecting the mediobasal temporal region and approached via endoscopic transorbital corridor by entering the temporal horn as intraoperatively confirmed by the egress of CSF. Authors performed the skull base reconstruction through abdominal fat graft intradurally placed, a multilayered technique using collagen matrix or acellular dermal matrix to cover the dural defect, additional fat graft extradurally placed to fill the dead space, and finally, fibrin glue to secure all the previous materials. Although the surgical corridor crossed the CSF pathway, with clear intraoperative observation of CSF leakage from the temporal ventricle horn, no cases of CSF leak were observed in the postoperative period.

The repositioning of the eyeball at the end of the procedure is also matter of debate: our group, in agreement with Moe [26], always used to leave 2–3 mm of proptosis considering the common resolution of the edema over the next 2 weeks which will lead to binocular symmetry; conversely other authors [25] repositioned the eyeball in its original position.

It is worth remembering that the transorbital pathway presents several intrinsic anatomical advantages: the eyeball functions as a vascularized tissue layer that provides a natural barrier which blocks the CSF leak; in addition, the bony defect resulting by drilling LOW is quite regular, roughly trapezoidal in shape, thus the reconstruction material easily fits the bony defect. Finally, the transorbital approach does not work against gravity, as the endonasal approach does, which simplifies reconstruction and further reduces the risk of CSF leakage.

In the present surgical series, our 4 F reconstruction technique was the same regardless the location and the size and volume of the osteodural defect because the intrinsic rationale behind the strategy was always the same. Intradural fat, in addition to being biocompatible, can be easily molded to fit dural defects different in shapes and sizes, creating a natural and soft barrier between brain and dura mater. Extradural fascia lata provides a strong, impenetrable and durable further barrier acting as a patch. A thin layer of fibrin glue is sprayed over to ensure fascia lata adhere to the dura mater. Finally, extradural fat between the orbit medially, the temporal pole dura mater posteriorly and the temporalis muscle laterally, fills the dead space without disrupting the adjacent structures. Care must be taken to avoid an overpacking which could account for proptosis.

This simple method of reconstruction allowed us to achieve no cases of postoperative CSF leak nor enophthalmos, however, this finding is based on a Limited sample size. One Limitation of the 4F technique is that, depending on the patient, one or two additional skin incisions—approximately 2 to 2.5 cm each—may be required. In patients with a higher BMI and consequently more abundant subcutaneous fat, sufficient fat and fascia lata can be harvested from the lateral thigh. However, in leaner patients, it is necessary to harvest abdominal fat in addition to fascia lata from the lateral thigh surface. In our series, only five patients required a second skin incision.

To overcome the potential complications related to the lack of bone resulting from LOW drilling, we proposed, through a laboratory anatomical-morphometric quantitative study, a piezoelectric orbitotomy as a method for establishing the surgical corridor preserving the lateral orbital wall [6].

### Limitation of the study

This study is limited by its retrospective design, small sample size, and case heterogeneity. Furthermore, no control group or comparative analysis with existing reconstruction techniques was included. As a result, claims regarding safety, effectiveness, and ease of application cannot be substantiated based on the current data. Given the low expected incidence (< 10%) of some complications, a significantly larger cohort would be necessary to draw reliable conclusions regarding safety and efficacy.

## Conclusion

The 4 F technique for the reconstruction of the osteodural defect after endoscopic transorbital surgery demonstrated promising results in achieving watertight closure and minimizing dead space, potentially contributing to the prevention of postoperative CSF leaks and enophthalmos However, in the absence of a control group, standardized outcome metrics, and considering the limited number of cases, no definitive conclusions regarding safety and efficacy can be drawn. Larger, prospective studies with comparative methods are required to validate these preliminary findings.

## Data Availability

No datasets were generated or analysed during the current study.

## References

[1] Bernardo A, Evins AI, Corvino S (2023) Microsurgical Anatomy of the Superior and Inferior Orbital Fissures. In: Springer C (ed) Cranio-Orbital Mass Lesions. In: Bonavolontà, G., Maiuri, F., Mariniello, G. (eds) 10.1007/978-3-031-35771-8_3

[2] Cappabianca P, Esposito F, Magro F, Cavallo LM, Solari D, Stella L, de Divitiis O (2010) Natura abhorret a vacuo–use of fibrin glue as a filler and sealant in neurosurgical “dead spaces.” Technical note. Acta Neurochir (Wien) 152:897–904. 10.1007/s00701-009-0580-2 20049488 10.1007/s00701-009-0580-2

[3] Cavallo LM, Solari D, Somma T, Cappabianca P (2019) The 3F (fat, flap, and flash) technique for skull base reconstruction after endoscopic endonasal suprasellar approach. World Neurosurg 126:439–446. 10.1016/j.wneu.2019.03.125 30904811 10.1016/j.wneu.2019.03.125

[4] Corvino S, Armocida D, Offi M, Pennisi G, Burattini B, Mondragon AV, Esposito F, Cavallo LM, de Notaris M (2023) The anterolateral triangle as window on the foramen lacerum from transorbital corridor: anatomical study and technical nuances. Acta Neurochir (Wien). 10.1007/s00701-023-05704-5 37479917 10.1007/s00701-023-05704-5 PMC10477108

[5] Corvino S, Berardinelli J, Corazzelli G, Altieri R, Dallan I, Corrivetti F, de Notaris M (2025) Surgical risk of CSF leakage following endoscopic transorbital approach for anterior and middle skull base pathologies: a systematic review and meta-analysis. Neurosurg Rev 48:282. 10.1007/s10143-025-03426-z 40044979 10.1007/s10143-025-03426-z PMC11882707

[6] Corvino S, de Notaris M, Sommer D, Kassam A, Kong DS, Piazza A, Corrivetti F, Cavallo LM, Iaconetta G, Reddy K (2024) Assessing the feasibility of selective piezoelectric osteotomy in transorbital approach to the middle cranial fossa: anatomical and quantitative study and surgical implications. World Neurosurg. 10.1016/j.wneu.2024.09.066 39303974 10.1016/j.wneu.2024.09.066

[7] Corvino S, Kassam A, Piazza A, Corrivetti F, Esposito F, Iaconetta G, de Notaris M (2024) Navigating the Intersection Between the Orbit and the Skull Base: The “Mirror” McCarty Keyhole During Transorbital Approach: An Anatomic Study With Surgical Implications. Oper Neurosurg. 10.1227/ons.0000000000001274 38995028 10.1227/ons.0000000000001274

[8] Corvino S, Kassam A, Piazza A, Corrivetti F, Spiriev T, Colamaria A, Cirrottola G, Cavaliere C, Esposito F, Cavallo LM, Iaconetta G, de Notaris M (2024) Open-door extended endoscopic transorbital technique to the paramedian anterior and middle cranial fossae: technical notes, anatomomorphometric quantitative analysis, and illustrative case. Neurosurg Focus 56:E7. 10.3171/2024.1.FOCUS23838 38560942 10.3171/2024.1.FOCUS23838

[9] Corvino S, Sacco M, Somma T et al (2023) Functional and clinical outcomes after superior eyelid transorbital endoscopic approach for spheno-orbital meningiomas: illustrative case and literature review. Neurosurg Rev 46:17 10.1007/s10143-022-01926-w 36513789

[10] Corvino S, Villanueva-Solórzano P, Offi M, Armocida D, Nonaka M, Iaconetta G, Esposito F, Cavallo L (2023) De notaris m (2023) a new perspective on the cavernous sinus as seen through multiple surgical corridors: anatomical study comparing the transorbital, endonasal, and transcranial routes and the relative coterminous spatial regions. Brain Sci 13(8):1215. 10.3390/brainsci13081215 37626571 10.3390/brainsci13081215 PMC10452901

[11] Dallan I, Sellari-Franceschini S, Turri-Zanoni M, de Notaris M, Fiacchini G, Fiorini FR, Battaglia P, Locatelli D, Castelnuovo P (2018) Endoscopic transorbital superior eyelid approach for the management of selected spheno-orbital meningiomas: preliminary experience. Oper Neurosurg 14:243–251. 10.1093/ons/opx100 29462449 10.1093/ons/opx100

[12] de Notaris M, Kong DS, Di Somma A, Enseñat J, Hong CK, Moe K, Schwartz TH (2024) Superior eyelid transorbital approaches: a modular classification system. J Neurosurg 141(1):278. 10.3171/2024.1.JNS232465 38626472 10.3171/2024.1.JNS232465

[13] de Notaris M, Sacco M, Corrivetti F, Grasso M, Corvino S, Piazza A, Kong DS, Iaconetta G (2023) The transorbital approach, a game-changer in neurosurgery: a guide to safe and reliable surgery based on anatomical principles. J Clin Med. 10.3390/jcm12206484 37892624 10.3390/jcm12206484 PMC10607762

[14] Di Somma A, Guizzardi G, Sanchez España JC, Matas Fassi J, Topczewski TE, Ferres A, Mosteiro A, Reyes L, Tercero J, Lopez M, Alobid I, Enseñat J (2023) Complications of the superior eyelid endoscopic transorbital approach to the skull base: preliminary experience with specific focus on orbital outcome. J Neuroophthalmol. 10.1097/WNO.0000000000001899 37410915 10.1097/WNO.0000000000001899

[15] Di Somma A, Kong DS, de Notaris M, Moe KS, Sánchez España JC, Schwartz TH, Enseñat J (2022) Endoscopic transorbital surgery levels of difficulty. J Neurosurg 137(4):1187. 10.3171/2022.3.JNS212699 35426817 10.3171/2022.3.JNS212699

[16] Hadad G, Bassagasteguy L, Carrau RL, Mataza JC, Kassam A, Snyderman CH, Mintz A (2006) A novel reconstructive technique after endoscopic expanded endonasal approaches: vascular pedicle nasoseptal flap. Laryngoscope 116:1882–1886. 10.1097/01.mlg.0000234933.37779.e4 17003708 10.1097/01.mlg.0000234933.37779.e4

[17] Hardesty DA, Montaser A, Kreatsoulas D, Shah VS, VanKoevering KK, Otto BA, Carrau RL, Prevedello DM (2021) Complications after 1002 endoscopic endonasal approach procedures at a single center: lessons learned, 2010–2018. J Neurosurg 1–12. 10.3171/2020.11.JNS202494 10.3171/2020.11.JNS202494 PMC10193480 34359021

[18] Jeon C, Hong CK, Chong K, Lee WJ, Kim GJ, Lee JI, Nam DH, Seol HJ, Choi JW, Shin HJ, Kong DS (2025) Endoscopic transorbital approach for resection of mediobasal temporal lesions using an optic radiation-sparing strategy. J Neurosurg 142:819–828. 10.3171/2024.6.JNS232810 39454213 10.3171/2024.6.JNS232810

[19] Jeon C, Hong CK, Woo KI, Hong SD, Nam DH, Lee JI, Choi JW, Seol HJ, Kong DS (2018) Endoscopic transorbital surgery for Meckel's cave and middle cranial fossa tumors: surgical technique and early results. J Neurosurg 1–10. 10.3171/2018.6.JNS181099 10.3171/2018.6.JNS181099 30544350

[20] Jeon C, Hong SD, Woo KI, Seol HJ, Nam DH, Lee JI, Kong DS (2020) Use of endoscopic transorbital and endonasal approaches for 360° circumferential access to orbital tumors. J Neurosurg 135(1):103. 10.3171/2020.6.JNS20890 32977310 10.3171/2020.6.JNS20890

[21] Kim JH, Hong CK, Shin HJ, Kong DS (2024) Feasibility and efficacy of endoscopic transorbital optic canal decompression for meningiomas causing compressive optic neuropathy. J Neurosurg 140:412–419. 10.3171/2023.5.JNS2326 37542442 10.3171/2023.5.JNS2326

[22] Kong DS, Kim YH, Hong CK (2020) Optimal indications and limitations of endoscopic transorbital superior eyelid surgery for spheno-orbital meningiomas. J Neurosurg 134:1472–1479. 10.3171/2020.3.JNS20297 32502989 10.3171/2020.3.JNS20297

[23] Lim J, Sung KS, Kim W, Yoo J, Jung IH, Choi S, Lim SH, Roh TH, Hong CK, Moon JH (2021) Extended endoscopic transorbital approach with superior-lateral orbital rim osteotomy: cadaveric feasibility study and clinical implications (SevEN-007). J Neurosurg 1–14. 10.3171/2021.7.JNS21996 10.3171/2021.7.JNS21996 34767525

[24] Maghalashvili E, Corrivetti F, Shalamberidze B, Corvino S, Chkhikvishvili T, de Notaris M (2024) Endoscopic transorbital resection of temporal pole cavernoma: 2-dimensional operative video. Oper Neurosurg. 10.1227/ons.0000000000001278 38967457 10.1227/ons.0000000000001278

[25] Mathios D, Bobeff EJ, Longo D, Nilchian P, Estin J, Schwartz AC, Austria Q, Anand VK, Godfrey KJ, Schwartz TH (2024) The lateral transorbital approach to the medial sphenoid wing, anterior clinoid, middle fossa, cavernous sinus, and Meckel’s cave: target-based classification, approach-related complications, and intermediate-term ocular outcomes. J Neurosurg 140:677–687. 10.3171/2023.6.JNS23678 37657097 10.3171/2023.6.JNS23678

[26] Moe KS (2024) Reconstruction and postoperative care after transorbital skull base and brain surgery. In: Schwartz TH, Kong DS, Moe KS (eds) Endoscopic transorbital surgery of the orbit, skull base and brain. Springer, Cham. 10.1007/978-3-031-59504-2_36

[27] Moe KS, Bergeron CM, Ellenbogen RG (2010) Transorbital neuroendoscopic surgery. Neurosurgery 67:ons16–28. 10.1227/01.NEU.0000373431.08464.43 10.1227/01.NEU.0000373431.08464.43 20679952

[28] Noiphithak R, Yanez-Siller JC, Revuelta Barbero JM, Otto BA, Carrau RL, Prevedello DM (2019) Comparative analysis between lateral orbital rim preservation and osteotomy for transorbital endoscopic approaches to the cavernous sinus: an anatomic study. Oper Neurosurg (Hagerstown) 16:86–93. 10.1093/ons/opy054 10.1093/ons/opy054 29701856

[29] Park HH, Hong SD, Kim YH, Hong CK, Woo KI, Yun IS, Kong DS (2020) Endoscopic transorbital and endonasal approach for trigeminal schwannomas: a retrospective multicenter analysis (KOSEN-005). J Neurosurg 133:467–476. 10.3171/2019.3.JNS19492 31226689 10.3171/2019.3.JNS19492

[30] Schwartz TH, Henderson F, Di Somma A, Kong DS, de Notaris M, Enseñat J, Moe KS (2022) Endoscopic transorbital surgery: another leap of faith? World Neurosurg 159:54–55. 10.1016/j.wneu.2021.12.081 35007891 10.1016/j.wneu.2021.12.081

[31] Tanoue Y, Uda T, Kawashima T, Yindeedej V, Goto T (2024) Endoscopic trans-orbital approach for the tumor-related epilepsy at the temporal tip. Neurosurgical Focus: Video 11:V9. 10.3171/2024.4.FOCVID2414 38957422 10.3171/2024.4.FOCVID2414 PMC11216418

